# Exploring the association of lifestyle behaviors and healthy ageing among the older adults in India: evidence from LASI survey

**DOI:** 10.1186/s12877-023-04367-2

**Published:** 2023-10-18

**Authors:** Raghunath Mandi, Dhananjay W Bansod, Amit Kumar Goyal

**Affiliations:** 1 International Institute for Population Sciences (IIPS), Mumbai, 400088 India; 2 Dept. of Public Health and Mortality Studies, International Institute for Population Sciences (IIPS), Mumbai, 400088 India

**Keywords:** Healthy ageing, Functional ability, Lifestyle behaviour, Physically active

## Abstract

**Background:**

Understanding health and developing trends among the older population is essential for countries to tackle the challenges of an ageing population and formulate relevant policies. Facilitating healthy ageing is an essential strategy to address the issues arising among the aged. The concept of healthy ageing is defined as “the process of developing and maintaining the functional ability that enables wellbeing in old age (WHO),“ where “functional ability comprises the health-related attributes that enable people to be and to do what they have reason to value.“ People have different ageing pathways depending on their genetic profile and different life course health risk exposures. Therefore, ageing, more specifically healthy ageing, largely depends on individual lifestyle choices. This study examines the association between lifestyle behaviours and healthy ageing among older adults in India.

**Methods:**

Based on the first round of LASI in 2017-18, we conceptualized healthy ageing within the WHO functional ability framework. We developed a Healthy Ageing Index (HAI), which incorporates physiological health, functional health, cognitive functions, psychological well-being, and social engagement. We used principal component analysis to generate a composite score for HAI. We then used multiple linear regression to demonstrate the association between lifestyle behaviours and HAI.

**Result:**

The mean HAI was 82.8%, indicating that the study population is healthier. The study findings show that smoking and drinking are more prevalent among males, rural residents, illiterate individuals, those currently employed, and those belonging to the poorest wealth quintile. Engaging in physical activity is associated with better health outcomes (β = 2.36; 95% CI: 2.16–2.56).

**Conclusion:**

This study emphasizes the importance of adopting a healthier lifestyle to achieve healthy ageing. Health behaviours are modifiable, so our results highlight the need for policy interventions to promote a healthier lifestyle from an early age.

**Supplementary Information:**

The online version contains supplementary material available at 10.1186/s12877-023-04367-2.

## Background

The increasing life expectancy resulting from medical and technological advancements, coupled with declining fertility rates has led to a significant increase in the number of older individuals worldwide. According to the latest population projection by World Population Prospects 2022, the proportion of individuals aged 65 and over is projected to rise from 10% to 2022 to 16% in 2050 [1]. However, living longer does not necessarily mean better health. Ageing has been associated with declining physical and cognitive functions and an increased risk of non-communicable diseases, which can lead to challenges in public health and healthcare resources [2]. Ageing pathways vary from person to person due to differences in genetic profiles and life course health exposures [3, 4]. It is therefore crucial to identify lifestyle behaviours that are associated with ageing.

The World Health Organization (WHO) defines healthy ageing as “the process of developing and maintaining the functional ability that enables wellbeing in old age” [5, 6]. Functional ability refers to people’s ability to meet their basic needs, learn, grow and make decisions, be mobile, build and maintain relationships, and contribute to society. Intrinsic capacity varies among individuals based on their genetic inheritance, physical and mental proficiency, and specific living and interacting environments. These environments can change over time depending on political, economic, social norms, values, and resources. The interaction between intrinsic capacity and environmental characteristics determines functional ability [7].

As individuals age, they lose functional abilities due to declining health and an increased risk of non-communicable diseases. Apart from that, exposure to different lifestyle behaviours might also affect health outcomes [8]. Age-related diseases could be delayed by adopting a healthy lifestyle. Regular engagement in physical activity and refraining from smoking can delay the ageing process [8].

Cigarette smoking is considered one of the major causes of death worldwide [9]. Studies have found an adverse relationship between smoking and functional ability, and a strong and consistent relationship between sustained smoking at age 50 to 70 and reduced functional ability at age 75, even after adjusting for physical activity [10, 11]. Furthermore, research suggests that smoking is positively associated with drinking alcohol and an unhealthy diet [12]. Study found that regular exercising and discontinuing smoking were significant predictors of good health, even after controlling for cardiovascular disease risk factors among older adults [13] and in the absence of smoking, diabetes, obesity, hypertension, or a sedentary lifestyle, it is 54% likely that lifespan at age 70 may increase to 90 years [14]. Early life exposure to healthy behaviours, including smoking abstinence and regular exercise, is associated with enhanced lifespan, good health, and function during old age [14]. Study found a positive association of limited alcohol consumption with healthy ageing [15].

There is ample evidence that behavioural factors notably smoking, diet, alcohol consumption and physical activity are associated with health and wellbeing of older adults. But very little evidence about its association with functional ability. In addition, most of the systematic reviews identified physiological health, physical capacities, cognitive functions, psychological and social wellbeing are most important domains associated with health ageing [16, 17]. In addition to lifestyle factors, healthy ageing differs across socioeconomic conditions. For instance, study in United State and England suggest financial status and educational attainment as strongest predictors of healthy ageing [18]. Because having more education spread more awareness and people to pay greater attention to their health. Similar association was found among the Chinese elderlies [18]. In addition, study suggests older people with disadvantaged socioeconomic positions are less likely to attain healthy ageing than elderlies from well-off socioeconomic conditions [19, 20]. Previous researchers also found that healthy ageing has not been equitable across population, mostly due to socioeconomic and demographic differentials [21]. Study among Indian older adults are noted higher vulnerability, primarily due to limited health and non-health entitlement. Therefore, investigating role of such factors are equally essential to come up with relevant policy interventions [22].

Based on the available literature, it is clear that there are multiple determinants of healthy ageing. Therefore, it is very relevant to identify various sociodemographic and modifiable risk factors which influence health and functional ability. However, there is no single agreed definition of how healthy ageing should be measured. Therefore, based on the available literatures including systematic reviews we identified important determinants of healthy ageing and tried to quantify the healthy ageing based on Longitudinal Ageing Study in India (LASI) data and see how it is associated with behavioural factors (including smoking, consuming alcohol and physical activity) among the older adults in India.

## Methods

### Data sources

This analysis is based on data from the first wave of the Longitudinal Ageing Study in India (LASI), a nationally representative survey on the health, social, and economic aspects of adults aged 45 and above across all states and union territories of India. The survey was conducted in the year 2017-18 [23]. LASI provides comprehensive information on demographics, household economic status, health conditions, healthcare access and utilization, family dynamics, and social security coverage.

To determine the predefined sample size, LASI adopted a multistage stratified area probability cluster sampling design. Rural areas were selected using a three-stage sampling design, while urban areas were selected using a four-stage sampling design. The first stage involved the selection of Primary Sampling Units (PSUs), which are sub-districts (Tehsils/Talukas), and in the second stage, villages for rural and wards for urban areas were chosen from the selected PSUs. In the third stage, households were selected from the selected villages. However, sampling in urban areas involved an additional stage. Specifically, in the third stage, a Census Enumeration Block (CEB) was randomly selected in each urban area, and in the fourth stage, households were selected from this CEB. The detailed sampling procedure is elaborated in the LASI report [23]. The LASI survey covered a sample size of 73,396 individuals aged 45 and above. However, our study focused on subjects aged 60 years and above, resulting in a sample size of 29,223 elders aged 60 years and above across all states and union territories of India.

### Outcome variable

In accordance with the WHO definition of healthy ageing, we conceptualize healthy ageing within the functional ability framework [5, 6]. We constructed Healthy Ageing Index (HAI) by considering 28 variables from multiple domains, including physiological health, functional health, cognitive functions, psychological wellbeing domains and social engagement. *Physiological health* was measured using absence of major chronic disease. We used nine important chronic diseases to identify the healthy agers including hypertension, diabetes, cancer, chronic lung disease, chronic heart disease, stroke, arthritis, neurological problem and cholesterol. The second component i.e., *functional health* defined by the physical capabilities of an individual, whether the respondents needed any assistance in activities of daily living (ADL) or instrumental activities of daily living (IADL) like dressing, walking, bathing, eating, getting out of bed, using toilet, cooking, shopping, making telephonic calls, taking medications, doing work around the house or garden, managing money, and getting around or finding address in unfamiliar place etc. The third component in healthy ageing is *Cognitive ability* using memory and orientation to time (date, month, and year). Fourth component being the *psychological wellbeing* measured by Centre for Epidemiologic Studies Depression Scale (CES-D). The original CES-D scale is a 20-item scale, while a shortened 10-item scale with four scale option categories was used in the LASI. The 10 items included seven negative symptoms (trouble concentrating, feeling depressed, low energy, fear of something, feeling alone, bothered by things, and everything is an effort), and three positive symptoms (feeling happy, hopeful, and satisfied). Response options included rarely or never (< 1 day), sometimes (1 or 2 days), often (3 or 4 days), and most or all of the time (5–7 days) in a week prior to the interview. The scoring was reversed for negative symptoms. For negative symptoms, rarely or never (< 1 day) were scored three, and sometimes (1 or 2 days) were scored two, often (3 or 4 days) were scored one, and most or all of the time (5–7 days) categories were scored zero. For positive symptoms rarely or never (< 1 day) were scored zero, and sometimes (1 or 2 days) were scored one, often (3 or 4 days) were scored two, and most or all of the time (5–7 days) categories were scored three [23]. The overall score ranges from zero to 30 and the score was further transformed to a quintile scale 0-100, with 0 represents no depressive symptoms 100 indicates healthy ager. The last component of Healthy Ageing Index is *social engagement*. The variable is measured with seven dimensions covering frequency of engagement in social activities like (1) go to park/beach for relaxing/entertainment, (2) play cards/indoor games, (3) play outdoor games/sports/yoga/exercise, (4) visit relatives/friends, (5) Attend cultural performances/shows/Cinema, (6) Attend religious functions/events such as bhajan/satsang/prayer, (7) Attend political/community/organization group meetings. Each of these questions have seven responses daily, several times a week, once a week, several times a month, at least once a month, rarely/once in a year, never. The overall score range from 0 to 28 and the score further transformed to a quintile scale 0–10. Each of the 28 variable was coded binary or quintile and then coded for the interval 0-100 (see Table S1 supplementary material). We used the principal component analysis (PCA) to create the composite score of Healthy Ageing Index (HAI) incorporating these 28 variables clubbed within these five domains i.e. physiological health, functional health, cognitive functions, psychological wellbeing domains and social engagement. The HAI score ranges from 0 to 100 with higher score indicated healthier ageing status.

The validity and reliability of the HAI was performed using Cronbach Alpha, and the full details are provided in the supplementary file. The Cronbach alpha was 0.83 indicates a good internal consistency. The eigenvalues and percentage of explained variance of the six factors of HAI score was shown in supplementary file table S3. Together these six factors accounts for 49.3% of the total variances of HAI (shown in supplementary file table S2).

### Predictor variables

#### Lifestyle behaviour measures

An individual’s lifestyle was measured with three health behaviours: current smoking, current drinking, and physical activity. Self-reported current smoking status was assessed with yes/no using the question on whether the respondents currently smoking (either smoked tobacco like cigarette, bidi, hookah, *chroot* or smokeless tobacco like chewing tobacco, *gutka*, pan masala etc.). Similarly, the alcohol history of the respondents was also studied and self-reported current drinking alcohol status was assessed with yes/no, using the question: over the past three month respondents had at least one alcoholic drink, for example-beer, wine or any drink.

WHO recommendation on moderate and vigorous physical activity were used for developing physical activity indicators [24]. Where *Moderate physical activity* has been defined by involvement of at least 150 min of moderate-intensity physical activity (such as cleaning house, washing clothes, fetching water, drawing water from a well, gardening, walking at a moderate pace, bicycling at a regular pace, and floor or stretching exercises) throughout the week. *Vigorous physical activity* is defined by involvement of at least 75 min of vigorous-intensity physical activity (like running or jogging, swimming, going to a health centre/gym, cycling, digging with a spade or shovel, heavy lifting, chopping, farm work, fast bicycling, and cycling with loads) throughout the week. Based on the response to moderate and vigorous physical activity, we classified respondents as *physically active* (those who are either engaged in moderate physical activity or vigorous physical activity or an equivalent combination of moderate- and vigorous-intensity activity) and *physically inactive* (those who are not engaged in any type of moderate or vigorous physical activity throughout the week) [23].

#### Socio-demographic measures

Age, sex, place of residence, marital status, educational attainment, working status, marital status, living arrangements, economic and social status etc. was taken as background variables. Age was categorized into three groups ‘60–69’, ‘ 70–79’, and ’80 and above’ to distinguish between life stages which are ‘youngest-old’, ‘middle-old’, and ‘oldest-old’ [25]. Sex was included as dichotomous variable; i.e., male or female. Place of residence was classified as rural or urban. Older adult’s educational levels were assessed using four categories ranging from ‘no schooling’, ‘less than 5 years’, ‘5–9 years’, and ’10 and more years of schooling’. Current marital status was classified into ‘currently married’ and ‘others’. Similarly, current working status as ‘yes’ and ‘no’. Economic status was indicated by household wealth quintile (‘poorest’, ‘poorer’, ‘middle’, ‘richer’ and ‘richest’). Living arrangements among the older adults were classified into ‘living alone or with spouse’ and ‘living with others’. Caste has been classified as ‘Scheduled Castes (SC)’, ‘Scheduled Tribes (ST)’, ‘Other Backward Classes (OBC)’, and ‘none of them’.

### Statistical analysis

Descriptive statistics were used in describing the characteristics of the older population in various socio-demographic conditions. The estimated prevalence of lifestyle behaviours (smoking, drinking and physical activity) and Healthy Ageing Index (HAI) were adjusted for age and sex fixed effect. Multiple linear regression analysis was performed with Healthy Ageing Index (HAI) as dependent variable and lifestyle behaviours and other socio-demographic covariates as independent variable. STATA 16.0 have been used to perform all the statistical analysis.

## Results

### Sociodemographic characteristics

The present analysis was carried out using 29,223 respondents who had responded to all the variable of interest for this study. In our study, approximately, 61% of the study population belong to the age group 60–69, females (52%), had no formal education (53%). Almost two third of the study population residing in rural areas (67%). Over 30% of the respondent were currently working. All the categories in MPCE quintile represented approximately equally. Around one third of the study population were currently smoking (31%), either smoked tobacco or smokeless tobacco and 10% were currently consuming alcohol. In terms of physical activity more than half were physically active (52%). Detailed description is given in Table 1.

**Table 1:** Sample profile of the study respondents

Socio-demographic Characteristics	Percentage	Frequency
Age Category		
60–69	61.3	17,908
70–79	28.8	8,410
80+	9.9	2,905
Sex		
Male	48.3	14,114
Female	51.7	15,109
Residence		
Rural	66.6	19,469
Urban	33.4	9,754
Education (in years)		
No Education	53.4	15,600
Less than 5	12.1	3,543
5–9	19.3	5,646
10 & more	15.2	4,434
Currently Working		
No	69.8	20,406
Yes	30.2	8,817
Currently Married		
No	35.9	10,484
Yes	64.1	18,739
Living Arrangement		
Living alone	5.1	1,475
Living with Spouse only	19.5	5,711
Living with others	75.4	22,037
MPCE Quintile		
Poorest	20.7	6,051
Poorer	20.6	6,014
Middle	20.5	5,986
Richer	19.7	5,760
Richest	18.5	5,412
Caste		
SC	16.7	4,894
ST	16.9	4,944
OBC	39.6	11,573
Others	26.8	7,812
Currently Smoking		
No	69.5	20,296
Yes	30.5	8,927
Currently Drinking		
No	90.5	26,429
Yes	9.5	2,794
Physical Activity		
Physically Inactive	48.0	14,036
Physically Active	52.0	15,187
Total	**100.0**	**29,223**

Table 2 shows the age-sex adjusted prevalence of lifestyle behaviours (smoking, drinking and physical activity) by socio-demographic characteristics. 31% of our study participants aged 60 and above are currently smoking. Smoking is more common among male (43.0; 95% CI: 42.2–43.8), residing in rural areas (35.6; 95% CI: 34.9–36.2), having no formal education (37.0; 95% CI: 36.2–37.7), currently working (38.6; 95% CI: 37.6–39.6), belongs to the poorest wealth quintile (34.5; 95% CI: 33.4–35.7). Similarly, the prevalence of drinking is more common among male (17.0; 95% CI: 16.4–17.7) age group 60–69 (10.7; 95% CI: 10.3–11.1) rural resident (11.1; 95% CI: 10.7–11.5) currently working (12.2; 95% CI: 11.6–12.8), belongs to ST category (16.5; 95% CI: 15.5–17.5), living alone (12.1; 95% CI: 10.0-14.1).

**Table 2:** Age-sex adjusted prevalence of lifestyle behaviour (smoking, drinking, and physical activity) by socio-demographic characteristics among the older adults in India, 2017-18

Socio-Demographic Characteristics	Smoking (95% CI) N = 29,223	Drinking (95% CI) N = 29,223	Physical Activity (95% CI) N = 29,223
Age Category			
60–69	31.5(30.9–32.2)	10.7(10.3–11.1)	58.6(57.9–59.3)
70–79	29.6(28.7–30.6)	8.5(8.0-9.1)	45.7(44.7–46.8)
80+	27.3(25.7–28.8)	5.7(4.9–6.6)	29.0(27.3–30.6)
Sex			
Male	43.0(42.2–43.8)	17.0(16.4–17.7)	48.0(47.1–48.8)
Female	18.9(18.3–19.6)	2.6(2.4–2.9)	55.7(54.9–56.5)
Residence			
Rural	35.6(34.9–36.2)	11.1(10.7–11.5)	53.0(52.3–53.7)
Urban	20.4(19.6–21.2)	6.4(5.9–6.9)	49.9(48.9–50.9)
Education (in years)			
No Education	37.0(36.2–37.7)	12.8(12.2–13.4)	50.8(50.0-51.6)
Less than 5	34.8(33.4–36.3)	9.3(8.4–10.2)	51.5(49.9–53.2)
5–9	27.0(26.0-28.1)	8.1(7.5–8.7)	53.8(52.5–55.0)
10 & more	14.7(13.8–15.6)	5.4(4.9–5.9)	54.1(52.6–55.6)
Currently Working			
No	26.7(26.1–27.3)	7.9(7.5–8.3)	45.0(44.4–45.7)
Yes	38.6(37.6–39.6)	12.2(11.6–12.8)	67.7(66.7–68.7)
Currently Married			
No	33.4(32.4–34.4)	10.7(9.9–11.5)	49.1(48.1–50.2)
Yes	29.2(28.6–29.9)	9.2(8.9–9.6)	53.5(52.8–54.3)
Living Arrangement			
Living alone	31.9(29.5–34.4)	12.1(10.0-14.1)	66.3(63.9–68.6)
Living with Spouse only	28.6(27.5–29.7)	10.3(9.6–11.0)	57.1(55.9–58.4)
Living with others	31.0(30.4–31.6)	9.2(8.9–9.6)	49.7(49.0-50.3)
MPCE Quintile			
Poorest	34.5(33.4–35.7)	10.3(9.6–11.1)	52.0(50.8–53.2)
Poorer	34.2(33.0-35.3)	9.3(8.6–10.0)	52.0(50.8–53.3)
Middle	31.2(30.1–32.4)	9.1(8.4–9.9)	52.6(51.4–53.8)
Richer	28.8(27.7–29.9)	9.7(8.9–10.4)	52.1(50.9–53.4)
Richest	23.3(22.2–24.4)	9.3(8.6–10.1)	51.0(49.7–52.3)
Caste			
SC	37.6(36.3–38.9)	11.8(10.9–12.7)	52.7(51.3–54.0)
ST	34.5(33.2–35.8)	16.5(15.5–17.5)	48.6(47.3–50.0)
OBC	29.2(28.5–30.0)	8.5(8.0–9.0)	53.9(53.0-54.8)
Others	25.5(24.6–26.5)	5.3(4.8–5.8)	50.8(49.7–51.9)

Physical activity was more prevalent among females (55.7; 95% CI: 54.9–56.5) belongs to the age group 60–69 (58.6; 95% CI: 57.9–59.3), residing in rural areas (53.0; 95% CI: 52.3–53.7) living alone (66.3; 95% CI: 63.9–68.6) and currently working (67.7; 95% CI: 66.7–68.7).

**Fig. 1:**
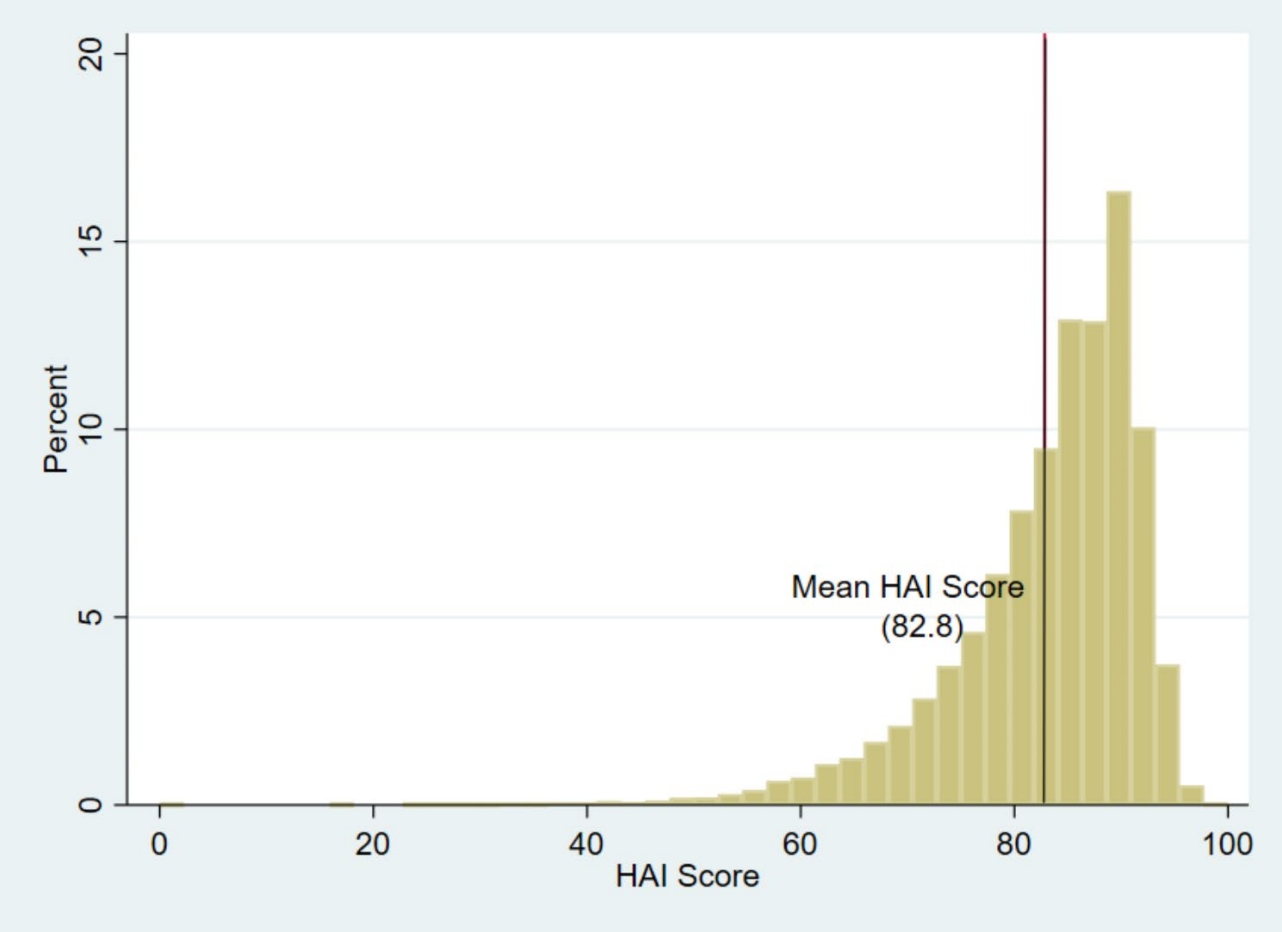
Distribution of healthy ageing score among the older adults in India

Figure 1. Shows the distribution of older adults in India based on the Healthy ageing score. The mean Healthy Ageing Index of our study population was 82.8%, representing the study population is healthier as shown in Fig. 1. A significantly larger share of our study sample (61%) in the age group 60 and above had healthy ageing score more than the mean HAI.

Table 3 shows age-sex adjusted score of healthy ageing among the older adults with exposure to various lifestyle behaviours (smoking, drinking, and physical activity) by socio-demographic characteristics in India. The score was adjusted for age and sex fixed effects. It was found that the exposure to smoking and drinking decline the healthy ageing with increasing age. Among the population with exposure to smoking, healthy ageing score was relatively better among male (84.7; 95% CI: 84.5–84.9), rural resident (84.7; 95% CI: 84.5–84.9), currently working (86.5; 95% CI: 86.2–86.7), and ST category (86.0; 95% CI: 85.6–86.3).

**Table 3:** Age-sex adjusted score of healthy ageing among the older adults with exposure to various lifestyle behaviour (smoking, drinking, and physical activity) by socio-demographic characteristics in India, 2017-18

Socio-demographic Characteristics	Smoking Mean HA Score (95% CI) (N = 8927)	Drinking Mean HA Score (95% CI) (N = 2937)	Physical Activity Mean HA Score (95% CI) (N = 16,163)
Age Category			
60–69	85.4(85.2–85.6)	85.9(85.5–86.2)	85.1(85.0-85.2)
70–79	83.5(83.2–83.8)	84.2(83.7–84.7)	83.7(83.5–83.9)
80+	80.7(80.1–81.2)	81.4(80.3–82.6)	83.1(82.5–83.6)
Sex			
Male	84.7(84.5–84.9)	85.1(84.8–85.4)	85.4(85.2–85.6)
Female	83.8(83.5–84.0)	85.8(85.0-86.5)	84.0(83.9–84.2)
Residence			
Rural	84.7(84.5–84.9)	85.5(85.2–85.8)	85.3(85.2–85.5)
Urban	83.5(83.1–83.8)	84.1(83.5–84.7)	83.2(83.0-83.4)
Education (in years)			
No Education	84.8(84.6–85.0)	85.9(85.5–86.3)	85.3(85.2–85.5)
Less than 5	84.5(84.1–84.9)	85.4(84.6–86.1)	84.2(83.8–84.5)
5–9	83.8(83.5–84.2)	83.7(83.1–84.3)	83.9(83.7–84.2)
10 & more	83.3(82.8–83.9)	84.5(83.7–85.3)	83.5(83.2–83.8)
Currently Working			
No	82.9(82.7–83.1)	83.4(83.0-83.8)	83.3(83.2–83.5)
Yes	86.5(86.2–86.7)	86.9(86.5–87.3)	86.6(86.4–86.8)
Currently Married			
No	83.8(83.5–84.2)	84.5(83.9–85.0)	83.7(83.5–83.9)
Yes	84.7(84.5–84.9)	85.4(85.1–85.7)	85.1(84.9–85.2)
Living Arrangement			
Living alone	84.1(83.3–84.9)	85.2(83.7–86.6)	83.4(82.9–83.8)
Living with Spouse only	84.8(84.4–85.1)	84.9(84.3–85.5)	84.8(84.5–85.0)
Living with others	84.3(84.2–84.5)	85.3(84.9–85.6)	84.7(84.6–84.8)
MPCE Quintile			
Poorest	85.1(84.8–85.5)	86.3(85.7–86.9)	85.5(85.2–85.8)
Poorer	84.9(84.6–85.3)	85.9(85.3–86.6)	85.2(84.9–85.4)
Middle	84.5(84.1–84.9)	85.1(84.5–85.8)	84.8(84.5–85.0)
Richer	84.1(83.7–84.4)	84.7(84.1–85.4)	84.3(84.1–84.6)
Richest	82.8(82.3–83.2)	83.6(82.9–84.2)	83.2(82.9–83.4)
Caste			
SC	84.3(83.9–84.7)	85.1(84.5–85.7)	84.8(84.5–85.1)
ST	86.0(85.6–86.3)	86.8(86.2–87.3)	87.0(86.7–87.3)
OBC	84.3(84.1–84.6)	84.5(84.0–85.0)	84.3(84.1–84.5)
Others	83.4(83.0-83.7)	83.8(83.1–84.6)	83.6(83.3–83.8)

While the healthy ageing score was higher among older adults belong to the age group 60–69 (85.9; 95% CI: 85.5–86.2) and ST population (86.8; 95% CI: 86.2–87.3), and currently working older adults (86.9; 95% CI: 86.5–87.3) who had exposure to drinking in past three months. Among the current smokers and alcohol consumers, healthy ageing score was relatively better among the older adults in poorest wealth quintile with no education. The healthy ageing score among the physically active participants is higher among the male (85.4; 95% CI: 85.2–85.6), those belongs to age group 60–69 (85.1; 95% CI: 85.0-85.2), currently working (86.6; 95% CI: 86.4–86.8), residing in rural areas (85.3; 95% CI: 85.2–85.5), and belongs to the ST category (87.0; 95% CI: 86.7–87.3).

Table 4 shows results of regression model showing relation between lifestyle behaviours (physical activity, smoking, and drinking) and sociodemographic characteristics on healthy ageing among the older adults in India aged 60 years and above. The regression analysis indicates a positive effect of physical activity on healthy ageing (β = 2.36; 95% CI: 2.16–2.56). There was a negative effect of age, sex(female), residence, educational attainment and working status. Compare to the male older adults, females were less likely to attain healthy ageing (β=-0.51; 95% CI: -0.75–0.27). Older adults in the oldest-old age group (80+) were less likely to attain healthy ageing in compared to those in the age group 60–70 (β=-2.63; 95% CI: -2.98–2.28). Among the social groups, older adults from the ST category had higher healthy ageing status (β = 2.07; 95% CI: 1.74–2.40). Working status among the older adults were protective factors in healthy ageing. Currently working older adults were more likely to attain healthy ageing in compared to those not currently working (β = 2.85; 95% CI, 2.62–3.08). Contrary to the conventional perception that “money can buy you happiness” our study found that older adults in the wealthiest quintile i.e., richer and richest were less likely to attain healthy ageing in compared to those in the poorest wealth quintile. Similarly, marital status was also associated with higher healthy ageing status. Older adults currently in marital union were more likely to attain healthy ageing than those not in marital union (β = 0.76; 95% CI, 0.51-1.00).

**Table 4:** Multiple linear regression of the potential factors associated with Healthy ageing among the older adults in India, 2017-18

Factors	β	95% CI	P > t
Physical Activity			
Physically Inactive®			
Physically Active	2.36	(2.16–2.56)	< 0.001
Currently Smoking			
No®			
Yes	0.64	(0.42–0.86)	< 0.001
Currently Drinking			
No®			
Yes	0.50	(0.16–0.84)	0.004
Age (in years)			
60–69®			
70–79	-1.15	(-1.38–0.93)	< 0.001
80+	-2.63	(-2.98–2.28)	< 0.001
Sex			
Male®			
Female	-0.51	(-0.75–0.27)	< 0.001
Place of Residence			
Rural®			
Urban	-1.04	(-1.26–0.82)	< 0.001
Education (in years)			
No education®			
less than 5 years	-0.97	(-1.28–0.66)	< 0.001
5–9 years	-1.01	(-1.28–0.74)	< 0.001
10 and more	-0.60	(-0.93–0.27)	< 0.001
Currently Working			
No®			
Yes	2.85	(2.62–3.08)	< 0.001
Currently Married			
No®			
Yes	0.76	(0.51-1.00)	< 0.001
Living Arrangements			
Living Alone®			
Living with spouse only	-0.20	(-0.72-0.32)	0.445
Living with others	-0.09	(-0.54-0.37)	0.714
MPCE Quintile			
Poorest®			
Poorer	-0.03	(-0.33-0.26)	0.827
Middle	-0.24	(-0.54-0.06)	0.115
Richer	-0.68	(-0.98–0.38)	< 0.001
Richest	-1.66	(-1.98–1.34)	< 0.001
Caste			
SC®			
ST	2.07	(1.74–2.40)	< 0.001
OBC	-0.25	(-0.53-0.03)	0.079
Others	-0.19	(-0.50-0.12)	0.218

**®** *Reference Category*

## Discussion

Drawing data from nationally-representative sample of the ageing population in India, we constructed Healthy Ageing Index (HAI), as per the WHO definition of *‘Healthy Ageing’* based on functional ability framework [5] comprising 28 variables from five major domains (i.e., psychological health, functional capabilities, cognitive ability, psychological wellbeing, and social well-being) [16] and then examine the relationships between behavioural characteristics (such as smoking, drinking, and physical activity) and healthy ageing among the older adults in India. The findings of our study are consistent with the previous studies indicating healthy ageing in old age is determined by various processes and lifestyle behaviours [4, 26–29]. The mean Healthy Ageing Index of our study population was 82.8 out of 100, representing the study population is healthier and a considerably higher percentage of our study sample (61%) had a healthy ageing score that was higher than the mean HAI.

Our findings regarding the lifestyle behaviour shows that the prevalence of smoking and drinking decrease with increasing age. However, its prevalence is more common among male, rural resident, illiterate, currently working, and those belongs to the poorest wealth quintile. In addition, we found a positive association between current smoking and alcohol consumption with healthy ageing. Although, these findings should be interpreted with causation as there are contradictory findings regarding the beneficial effect of limited alcohol consumption [15, 26, 30–32]. Due to the mixed evidence available in the existing literature regarding the effect of smoking and drinking, future studies are needed to see the effect of frequency and duration of consumption of tobacco and alcohol on healthy ageing, which may provide a new insight.

Our study highlighted the role of health promoting behaviour i.e., physical activity on healthy ageing among the older adults and confirmed its strong association on the experience of healthy ageing [27, 32]. The complex association between physical activity and better health status had been widely studied. Older adults with higher physical activity have better metabolism, higher cell endurance, muscle tissue functionality and energy metabolism and reduced age associated neurodegenerative disorders [33]. Older adults who either engaged in moderate or vigorous physical activity have higher functional ability. Older adults those having more social participation through interaction with outside people, participating in social activities have more chances to be a healthy person. This findings is consistent with other studies showing similar association [4, 27, 28, 32].

In addition to the lifestyle habits, healthy ageing differs by the sociodemographic characteristics of the study population. The present study revealed an inverse relation of healthy ageing with increasing age. According to the results of recent research among Indian elderlies, it was found that an individual’s risk of developing several chronic diseases and disabilities rises as they become older [34], which may negatively impact on their intrinsic capacity and consequently on functional ability [2, 34–36]. Therefore, it is not surprising that age has been negatively associated with healthy ageing.

We found significant relationship between gender and health behaviour patterns. Similar to the previous studies [27, 37, 38] our results also shows that females were less likely to smoke and consume alcohol than men and more likely to engaged in physical activities. Our results coincide with previous findings that female older adults had lower level of healthy ageing than male older adults [22]. This could be explained due to the higher prevalence of multi-morbidity reported in females compared to males of the same age [34]. However, this association is more influenced by age than sex, especially after age 80, due to the declining health status with advancing age [36]. In addition, a strong positive association of healthy ageing status was observed among older adults from Scheduled Tribe community and those currently working. However, studies examining these relationships are scarce in the literature and needs to be further investigated. Therefore, our results suggest marked differentials in socio-demographic factors associated with healthy ageing among older adults in India. .

With increasing age, people may encounter with number of chronic diseases [39–42] and declining functional ability [43], and some of them may last for even decades. Sometimes people are “used to” live with these diseased conditions. Therefore, healthy ageing does not mean older people without disease, but to live with the diseased conditions healthily with good functional ability.

Based on our knowledge, this study is among the first ones employed the functional ability framework to operationalize the healthy ageing in accordance with WHO framework [5] and shows association with lifestyle behaviours among the older adults in India. Our results contribute to the advance healthy ageing knowledge base which opens a horizon for future research in this domain. But our findings should be interpreted within the context of various study limitations. First, this study is based on cross-sectional survey that limits the possibility for causal relationships. Second, the lifestyle behaviours i.e., current smoking status and current alcohol consumption status were assessed via self-reported questionnaire. Therefore, potential measurement errors could have been occurred. In addition, our analysis focused on older adults aged 60 years and above without considering their early life exposure to these lifestyle behaviours. However previous research also found that early life factors and exposures to lifestyle behaviours may have impact on the health outcome in old age [15, 44]. Although the research is purely data-driven and depends only on the first round of Longitudinal Ageing Study in India (LASI), future research should focus on replicating these findings including other subpopulations which will contribute to life course perspective of health outcomes in old age.

## Conclusion

In conclusion, the present study highlights the importance of promoting healthier lifestyle for better health outcome among the older population in India. Physical activity was significantly associated with healthy ageing status among the older population. In addition, the present study also highlights the socio-demographic inequalities in healthy ageing. As these health behaviours are modifiable, our results highlight the need for health policy interventions to promote the healthier lifestyle from early ages.

### Electronic supplementary material

Below is the link to the electronic supplementary material.

Supplementary Material 1

## Data Availability

The study uses secondary data which is available on reasonable request through https://www.iipsindia.ac.in/content/LASI-data.

## List of abbreviations

HAI: Healthy Ageing Index
SC: Scheduled Castes
ST: Scheduled Tribes
OBC: Other Backward Classes
MPCE: Monthly Per Capita Consumption Expenditure
ADL: Activities of Daily Living
LASI: Longitudinal Ageing Study in India
