# Supplementary material for: Exploring the association of lifestyle behaviors and healthy ageing among the older adults in India: evidence from LASI survey
Source: BMC Geriatr. 2023 Oct 18;23:675. doi: 10.1186/s12877-023-04367-2 (PMC10585826; doi:10.1186/s12877-023-04367-2)
Supplement: Supplementary file 1 — Supplementary Material 1 [file 12877_2023_4367_MOESM1_ESM.docx]

**Supplementary file: Healthy Ageing Index**

**Table S1:** Healthy ageing indicators based on Functional Ability (WHO Framework)

| **Domains** | | **Variable** | **Categories** | **Scale** |
| --- | --- | --- | --- | --- |
| Chronic Disease (Physiological Health) | | Hypertension | 1= Yes and 0= No | 1 = 0 and 0= 100 |
|  |  | Diabetes | 1= Yes and 0= No | 1 = 0 and 0= 100 |
|  |  | Cancer | 1= Yes and 0= No | 1 = 0 and 0= 100 |
|  |  | Chronic Lung Disease | 1= Yes and 0= No | 1 = 0 and 0= 100 |
|  |  | Chronic Heart Disease | 1= Yes and 0= No | 1 = 0 and 0= 100 |
|  |  | Stroke | 1= Yes and 0= No | 1 = 0 and 0= 100 |
|  |  | Arthritis | 1= Yes and 0= No | 1 = 0 and 0= 100 |
|  |  | Neurological Problem | 1= Yes and 0= No | 1 = 0 and 0= 100 |
|  |  | High Cholesterol | 1= Yes and 0= No | 1 = 0 and 0= 100 |
| Physical capabilities  (Functional Health) | ADL | Dressing | 1= Yes and 0= No | 1 = 0 and 0 = 100 |
|  |  | Walking | 1= Yes and 0= No | 1 = 0 and 0 = 100 |
|  |  | Bathing | 1= Yes and 0= No | 1 = 0 and 0 = 100 |
|  |  | Eating | 1= Yes and 0= No | 1 = 0 and 0 = 100 |
|  |  | Getting out of bed | 1= Yes and 0= No | 1 = 0 and 0 = 100 |
|  |  | Using toilet | 1= Yes and 0= No | 1 = 0 and 0 = 100 |
|  | IADL | Cooking | 1= Yes and 0= No | 1 = 0 and 0 = 100 |
|  |  | Shopping | 1= Yes and 0= No | 1 = 0 and 0 = 100 |
|  |  | Making telephone calls | 1= Yes and 0= No | 1 = 0 and 0 = 100 |
|  |  | Taking medications | 1= Yes and 0= No | 1 = 0 and 0 = 100 |
|  |  | Doing work around the house or garden | 1= Yes and 0= No | 1 = 0 and 0 = 100 |
|  |  | Managing money | 1= Yes and 0= No | 1 = 0 and 0 = 100 |
|  |  | Getting around or finding address in unfamiliar place (mobility/movement) | 1= Yes and 0= No | 1 = 0 and 0 = 100 |
| Cognitive Functions/Ability* | | 10-word recall test (immediate recall) (mh011) | 0 – 10 | 0 = 0  1 = 10  2 = 20  3 = 30  4 = 40  5 = 50  6 = 60  7 = 70  8 = 80  9 = 90  10 = 100 |
|  |  | Orientation Time – date (mh002) | 1= correct and 0=incorrect | 1= 100 and 0 = 0 |
|  |  | Orientation Time – month (mh003) | 1= correct and 0=incorrect | 1= 100 and 0 = 0 |
|  |  | Orientation Time – year (mh004) | 1= correct and 0=incorrect | 1= 100 and 0 = 0 |
| Psychological wellbeing | | Center for Epidemiological Studies Depression (CES-D) | 0 -30 | 0-5 = 0  6-11 = 25  12-17 = 50  18 – 23 = 75  24 – 30 = 100 |
| Social Engagement/ Social Wellbeing | | Frequency of engagement in leisure time activities or organizing any social activities: (go to park/beach for relaxing/entertainment, play cards/indoor games, play outdoor games/sports/yoga/exercise, visit relatives/friends, attend cultural performances/shows/cinema, attend religious functions/events such as bhajan/satsang/prayer, attend political/community/organization group meetings | 0-28 | 0 = 0  1-7 = 25  8-14 = 50  15-21 = 75  22-28 = 100 |
| The total score of 28 variables has been normalized in to 0-100 scale | | | | |

*Original *Cognitive Composite Index* consisting 43 items (*memory, orientation, retrieval fluency, arithmetic functions, executive functions, objective naming*). We are considering only the *memory* and *orientation*) to capture the cognitive ability.

**Reliability and Validity of HAI**

**Internal Consistency of HAI**

- **Cronbach Alpha = 0.837**

**Table S2:** Eigen values and percentage of explained variance for Healthy Ageing Index (HAI)

| Components | Eigen value | Percentage Explained Variance |
| --- | --- | --- |
| Component 1 | 6.24299 | 22.3 |
| Component 2 | 2.39831 | 8.57 |
| Component 3 | 1.62226 | 5.79 |
| Component 4 | 1.45487 | 5.2 |
| Component 5 | 1.07188 | 3.83 |
| Component 6 | 1.00754 | 3.6 |

*Component 1 and component 2 are the major factor explaining the variance of HAI. Together, these six component account for 49.3% of the variance of HAI.

**Table S3:** Principal Component Analysis results for Healthy Ageing Index (HAI)

| Variable | PC1 | PC2 | PC3 | PC4 | PC5 | PC6 |
| --- | --- | --- | --- | --- | --- | --- |
| Physiological Health |  |  |  |  |  |  |
| Hypertension | 0.0342 | 0.2012 | 0.2728 | 0.394 | -0.1182 | -0.0362 |
| Diabetes | 0.0013 | 0.2204 | 0.2392 | 0.3287 | -0.2308 | -0.0542 |
| Cancer | 0.0085 | 0.0422 | 0.0476 | 0.0579 | -0.0016 | 0.6349 |
| Chronic Lung Disease | 0.0312 | 0.0219 | 0.0833 | 0.1322 | 0.4452 | -0.1777 |
| Chronic Heart Disease | 0.0145 | 0.1479 | 0.1883 | 0.3056 | -0.079 | -0.0171 |
| Stroke | 0.0665 | 0.1065 | 0.0211 | 0.1746 | -0.1826 | 0.3049 |
| Arthritis | 0.0683 | 0.0713 | 0.0805 | 0.2145 | 0.5215 | -0.2369 |
| Neurological Problem | 0.056 | 0.035 | 0.0348 | 0.1582 | 0.5136 | 0.2421 |
| High Cholesterol | 0.002 | 0.1787 | 0.2025 | 0.3528 | -0.1527 | -0.0443 |
| Functional Health |  |  |  |  |  |  |
| Dressing | 0.248 | 0.2015 | -0.2715 | 0.0145 | -0.0617 | 0.0284 |
| Walking | 0.2617 | 0.2047 | -0.2712 | 0.0073 | -0.0457 | -0.0064 |
| Bathing | 0.2686 | 0.2195 | -0.27 | 0.0103 | -0.0537 | -0.0085 |
| Eating | 0.2355 | 0.1639 | -0.204 | -0.0357 | -0.0546 | -0.0287 |
| Getting out of Bed | 0.2613 | 0.2041 | -0.1935 | 0.0194 | 0.0328 | -0.1151 |
| Using toilet | 0.2566 | 0.1849 | -0.1172 | 0.0017 | 0.0461 | -0.1488 |
| Cooking | 0.2734 | 0.0489 | 0.108 | -0.0982 | 0.0084 | -0.0342 |
| Shopping | 0.2929 | -0.0387 | 0.2261 | -0.1132 | -0.0071 | -0.0053 |
| Making telephonic calls | 0.2373 | -0.1902 | 0.2495 | -0.1296 | -0.0316 | 0.0249 |
| Taking medications | 0.2479 | -0.058 | 0.1614 | -0.1642 | -0.0645 | 0.0365 |
| Doing work around house/garden | 0.2825 | 0.0171 | 0.2057 | -0.0686 | 0.0289 | -0.0112 |
| Managing Money | 0.2761 | -0.1473 | 0.2922 | -0.1266 | -0.0211 | 0.0352 |
| Movement | 0.27 | -0.1358 | 0.2758 | -0.1193 | -0.0133 | 0.0273 |
| Cognitive Ability |  |  |  |  |  |  |
| 10 Word recall test | 0.1173 | -0.2701 | -0.1592 | 0.2104 | -0.0817 | 0.0797 |
| Date Orientation-day | 0.1375 | -0.3919 | -0.1407 | 0.2535 | -0.0768 | -0.119 |
| Date Orientation-month | 0.1188 | -0.3283 | -0.1404 | 0.3124 | -0.0717 | -0.1224 |
| Date orientation-year | 0.1384 | -0.4163 | -0.1324 | 0.2403 | -0.0398 | -0.0976 |
| Psychological Wellbeing |  |  |  |  |  |  |
| CESD | 0.1067 | -0.0418 | -0.0633 | 0.1029 | 0.3081 | 0.3602 |
| Social Engagement | 0.095 | -0.1027 | -0.1425 | 0.1375 | -0.0034 | 0.3661 |

**Robustness of Multiple Linear Regression**

**Table S4. Model Fitting Information**

| Number of Observation | 29,223 |
| --- | --- |
| Prob > F | 0.0000 |
| Adjusted R-Square | 0.1134 |

**Table S5:** Multiple linear regression of the potential factors associated with Healthy ageing among the older adults in India, 2017-18

| Factors | Coeff (β) | Std. Err | t | p> \| t \| | 95% CI | |
| --- | --- | --- | --- | --- | --- | --- |
| Physical Activity |  |  |  |  |  |  |
| Physically Inactive® |  |  |  |  |  |  |
| Physically Active | 2.359049 | 0.101709 | 23.19 | 0.000 | 2.159695 | 2.558404 |
| Currently Smoking |  |  |  |  |  |  |
| No® |  |  |  |  |  |  |
| Yes | 0.638199 | 0.113392 | 5.63 | 0.000 | 0.415947 | 0.860452 |
| Currently Drinking |  |  |  |  |  |  |
| No® |  |  |  |  |  |  |
| Yes | 0.500892 | 0.173986 | 2.88 | 0.004 | 0.159872 | 0.841911 |
| Age (in years) |  |  |  |  |  |  |
| 60-69® |  |  |  |  |  |  |
| 70-79 | -1.15448 | 0.113988 | -10.13 | 0.000 | -1.3779 | -0.93106 |
| 80+ | -2.63079 | 0.176881 | -14.87 | 0.000 | -2.97749 | -2.2841 |
| Sex |  |  |  |  |  |  |
| Male® |  |  |  |  |  |  |
| Female | -0.50799 | 0.122679 | -4.14 | 0.000 | -0.74844 | -0.26753 |
| Place of Residence |  |  |  |  |  |  |
| Rural® |  |  |  |  |  |  |
| Urban | -1.03614 | 0.112068 | -9.25 | 0.000 | -1.2558 | -0.81648 |
| Education (in years) |  |  |  |  |  |  |
| No education® |  |  |  |  |  |  |
| less than 5 years | -0.96633 | 0.158242 | -6.11 | 0.000 | -1.27649 | -0.65616 |
| 5-9 years | -1.00909 | 0.139404 | -7.24 | 0.000 | -1.28233 | -0.73585 |
| 10 and more | -0.59857 | 0.168198 | -3.56 | 0.000 | -0.92824 | -0.26889 |
| Currently Working |  |  |  |  |  |  |
| No® |  |  |  |  |  |  |
| Yes | 2.850847 | 0.118389 | 24.08 | 0.000 | 2.618798 | 3.082895 |
| Currently Married |  |  |  |  |  |  |
| No® |  |  |  |  |  |  |
| Yes | 0.759524 | 0.124841 | 6.08 | 0.000 | 0.51483 | 1.004218 |
| Living Arrangements |  |  |  |  |  |  |
| Living alone® |  |  |  |  |  |  |
| Living with spouse only | -0.20285 | 0.265671 | -0.76 | 0.445 | -0.72358 | 0.317877 |
| Living with others | -0.08559 | 0.233444 | -0.37 | 0.714 | -0.54315 | 0.371967 |
| MPCE Quintile |  |  |  |  |  |  |
| Poorest® |  |  |  |  |  |  |
| Poorer | -0.03305 | 0.151091 | -0.22 | 0.827 | -0.32919 | 0.263098 |
| Middle | -0.23997 | 0.152262 | -1.58 | 0.115 | -0.53841 | 0.058473 |
| Richer | -0.67965 | 0.155263 | -4.38 | 0.000 | -0.98397 | -0.37533 |
| Richest | -1.6558 | 0.162884 | -10.17 | 0.000 | -1.97506 | -1.33654 |
| Caste |  |  |  |  |  |  |
| SC® |  |  |  |  |  |  |
| ST | 2.071323 | 0.167604 | 12.36 | 0.000 | 1.742811 | 2.399834 |
| OBC | -0.25012 | 0.142374 | -1.76 | 0.079 | -0.52918 | 0.028935 |
| None of them | -0.19471 | 0.158043 | -1.23 | 0.218 | -0.50448 | 0.115065 |

**®***Reference Category*
